# Patterns of sports sponsorship by gambling, alcohol and food companies: an Internet survey

**DOI:** 10.1186/1471-2458-6-95

**Published:** 2006-04-11

**Authors:** Anthony Maher, Nick Wilson, Louise Signal, George Thomson

**Affiliations:** 1 Department of Public Health, Wellington School of Medicine and Health Sciences, Wellington, New Zealand

## Abstract

**Background:**

Sports sponsorship is a significant marketing tool. As such, it can promote products that pose risks to health (eg, high fat and high sugar foods) or it can promote health-supporting products (eg, sporting equipment and services). However, there is a lack of data on the proportion of sponsorship associated with "unhealthy" and "healthy" products and no methodology for systematically assessing it. This research aimed to explore this proportion with an Internet survey of sports sponsorship in the New Zealand setting.

**Methods:**

A search methodology was developed to identify Internet-based evidence of sports sponsorship at the national level and at the regional and club level in one specific region (Wellington). The top eight sports for 5-17-year-olds were selected and products and services of sponsors were classified in terms of potential public health impact (using a conservative approach).

**Results:**

Sponsorship of these popular sports was common at the national, regional and club levels (640 sponsors listed on 107 websites overall). Sports sponsorship associated with sponsors' products classified as "unhealthy" (eg, food high in fat and sugar, gambling and alcohol) were over twice as common as sponsorship associated with sponsors' products classified as "healthy" (32.7% (95% CI = 29.1, 36.5) versus 15.5% (95% CI = 12.8, 18.6) respectively). "Gambling" was the most common specific type of sponsorship (18.8%) followed by alcohol (11.3%).

There were significantly more "alcohol" sponsors for rugby, compared to all the other sports collectively (rate ratio (RR) = 2.47; 95% CI = 1.60, 3.79), and for top male sports compared to female (RR = 1.83; 95% CI = 1.05, 3.18). Also there was significantly more "unhealthy food" sponsorship for touch rugby and for "junior" teams/clubs compared to other sports collectively (RR = 6.54; 95% CI = 2.07, 20.69; and RR = 14.72, 95% CI = 6.22, 34.8; respectively). A validation study gave an inter-rater reliability for number of sponsors of 95% (n = 87 sponsors), and an inter-rater reliability of classification and categorisation of 100%.

**Conclusion:**

This study found that the sponsorship of popular sports for young people is dominated by "unhealthy" sponsorship (ie, predominantly gambling, alcohol and unhealthy food) relative to "healthy" sponsorship. Governments may need to consider regulations that limit unhealthy sponsorship and/or adopt alternative funding mechanisms for supporting popular sports.

## Background

Some of the key public health concerns in developed countries include alcohol misuse; poor nutrition – including excessive calories, saturated fat and free sugars; and gambling (given its association with problem gambling) [1-3]. The marketing of these products are therefore an area of concern from a public health perspective. Sports sponsorship appears to be becoming a prominent marketing tool used by companies, including those that promote alcohol, foods with poor nutritional value, and gambling. In the United States, sponsorship expenditures have increased from $850 million in 1985 to $8.5 billion in 2002 [4]. Compared to advertising, sponsorship is seen as inexpensive and is often more accepted by the public because it is more indirect and builds public goodwill towards the company [4]. Sponsorship is defined as "a cash and/or in kind fee paid to a property in return for access to the exploitable commercial potential associated with that property." Some of these commercial potentials of sports sponsorship include promotional opportunities, personal endorsements, and sometimes exclusive stocking agreements [5].

An important commercial benefit of sports sponsorship is that it associates sponsors products with healthy positive images, something that is particularly important for products that pose risks to health [6,7]. The association of a healthy activity (ie, sport) with such products obscures the health risk issue while at the same time promoting consumption [8]. In New Zealand sponsorship of sports by alcohol companies, foods with poor nutrition and gambling entities appears to be widespread. For example, a New Zealand brewery (Speights) supports rugby, netball, and multi-sport; while the fast food company McDonalds supports junior netball, touch rugby, and cricket. Gaming machine trusts are the legally required organisations that distribute funds from gaming/poker machines situated in New Zealand pubs and taverns. These trusts support nearly all popular sports and more recently have been beginning to acquire naming rights of many sporting tournaments, for example "the Scottwood Trust Netball Champs", and "the New Zealand Community Trust Soccer Championships".

However, sponsorship is not limited to products with potential health risks. Examples around the world (eg, in Australia), have shown that sponsorship programmes by health promotion organisations can lead to health and sporting organisations successfully collaborating [9]. In fact, sports sponsorship is seen as an ideal vehicle for health promotion companies, because it can access some hard-to-reach groups who participate in and support sport [6].

A recent New Zealand study examined sponsorship and fund-raising in New Zealand schools, and although there were some health promotion groups involved, there was a high proportion of sponsors with the potential for promoting products and activities that may threaten health (eg, gambling, alcohol and poor nutrition) [10]. Moreover, an American study in the late 1990s used an Internet-based method to describe the nature and extent of corporate sponsorship by tobacco companies [11]. In this study, we aimed to build on this previous work and examine the extent and nature of both "healthy" and "unhealthy" sport sponsorship for popular New Zealand sports. Furthermore, we aimed to design a methodology for monitoring sports sponsorship over time using an Internet-based method.

## Methods

### Sports and website selection

The sports were chosen to represent the most popular sports that young people in New Zealand participate in. The top five sports for boys and then girls aged 5–17 years were selected from participation figures supplied by the Sport and Recreation Council of New Zealand (SPARC) [12]. These were based on data from the 2001 New Zealand census and gave a total of eight sports. Rugby, cricket and touch rugby (a minimal contact form of rugby) were in the male top five sports; netball, athletics and tennis were in the female top five; and basketball and soccer were in both the female and the male top five.

Sponsorship information was obtained through examining national (ie, New Zealand wide), regional and club/team level websites. As this was a pilot study only regional and club organisations based in the Greater Wellington Region were examined. National and regional websites were found using a Google search for the words "New Zealand" or "Wellington" and the specific sport. Club websites were then found using the "club" links from the regional websites. For a club/team website to be included in the study it had to have its own exclusive webpage(s), contain the club name, and have some relevant club details or information available for the viewer. Websites that didn't work, didn't have their own page, or had no relevant information about the club for visitors were noted, but excluded from the study results.

### Data collection and validation

All pages of each website were examined in January 2006 by one of the authors (AM) to identify information about sponsorship or funding. A search for the word "sponsor" was used if the site had its own search engine. The search included any publications available on the website, for example newsletters or annual reports. We defined Internet-based indications of sports sponsorship as "companies or organisations that had one of the following reported on a team/club website: (i) naming rights of sports teams or clubs; (ii) being official sponsors or partners; (iii) sponsoring specific tournaments or scholarships; (iv) being involved in fundraising activities in the club; or (v) had the company logo on the team/club website." We excluded promotional material from the information technology companies that designed the various websites from this definition.

Various data collected included the brand, company or organisation type, primary product or service, location on the website, presence of a logo, presence of a link to the sponsor's website, and the presence of a description about the product or sponsorship agreement.

A sample of 10 websites (9.3% of the total) was independently examined by another observer. The inter-rater reliability of number for sponsors was 95% (based on n = 87 sponsors). However, for those sponsors that were the same, the inter-rater reliability for further classification and categorisation was 100%.

### Further categorization

Each sponsoring company or organisation was grouped into 11 categories, according to the primary product or service that they provided. Categories were: alcohol-related (including products or trusts), gambling-related (including trusts or similar entities), food products or companies, health promotion "products" (eg, smokefree messages), inactive entertainment (eg, television channels, cinemas, video stores), and non-active transport (eg, car companies), sporting goods companies, sporting organisations (eg, SPARC), sporting venues, other non-commercial organisations (eg, city councils) and other companies. As per the classification system in a recent New Zealand food advertising study [13], food categories were further classified as being: "healthy" if they were "favouring improved nutrition" (eg, fruit, vegetables, low sugar cereals); "unhealthy" if they were "counter to improved nutrition" (eg, foods high in fat and/or sugar, including fast food meals); or "mixed nutritional/health" profile (eg, juice, high fat milk, and meals with multiple components). Each company or organisation was also grouped according to their company structure or ownership to give the following categories: gambling trusts, franchises or multi-national companies, privately owned companies, city councils, government agencies, and non-government agencies.

A sponsor was defined as targeting a "junior" sport if the website was for a junior club or team, or if the sponsorship was for a junior grade or school-aged tournament.

### Health-related classification

A classification system for the potential impact on health of the products and services of the sponsoring companies was created. "Healthy" products and services included foods that were classified as "healthy" (see above), health promotion messages from health agencies (eg, smokefree messages), sporting organisations, sporting venues and sporting goods. "Unhealthy" products and services included alcohol, gambling, and "unhealthy foods". All other products and services were grouped into a "not classified" category. A conservative approach was taken so that sponsored products and services with mixed or ambiguous health aspects were placed in the "not classified" group (eg, foods with mixed nutritional characteristics, inactive-entertainment and non-active transport).

### Data analysis

All the data was entered into an Excel spreadsheet and analysed using the EpiInfo software package (CDC, Atlanta). OpenEpi was used to calculate statistically significant differences.

## Results

### Amount and location of sponsors and websites

Overall, 100% of national organisations, 85% of listed regional organisations and 45% of clubs listed by regional organisations had working websites (ie, were accessible and fitted into our definition of a website). This gave a total of 107 websites that met the criteria for inclusion in the study, however only 79 (73.8%) of these contained information about sponsorship. Of these 79, nine websites were national (11.4%), 12 were regional (15.2%) and 58 (73.4%) were at club level (Table 1). There were a total of 640 sponsors, giving an average of 8.2 sponsors per relevant website (ie, those containing sponsorship information). The range per relevant website was 1 to 43 sponsors. National levels had the highest average amount of sponsorship per team/website with 14.6 per relevant website, regional level had an average of 8.8 and club an average of 7.1. Rugby had the highest average number of sponsors with 12.8 per website, followed by touch rugby (9.5) and netball (9.2). Athletics had the lowest with 3.4 per website (Table 1).

The majority of sponsors were found on a specific "sponsors" page of the website (50.3%) or on the home page (32.2%). Of all the sponsors, 56.9% had their logo situated on club or team websites. Furthermore, 43.6% of all sponsors had links to their own homepage on the club or team websites.

Overall, there were 398 different sponsor companies and organisations. The most common sponsor (by number) was the New Zealand Community Trust, which sponsored 40 different teams/clubs, and accounted for 6.3% of all sponsorship. Within the top 10 most common sponsors, five were gaming machine trusts (Table 2).

### Characteristics of sponsorship

"Gambling" was the most common specific sponsorship category with 18.8% of the total (95% confidence interval (CI) = 15.8, 22.0). The majority of sponsorship in this category was by gaming machine trusts. "Alcohol" made up 11.3% (95% CI = 9.0, 14.0) of all sponsorship, and these were primarily bars and beer companies. "Sporting goods" made up 8.1% (95% CI = 6.2, 10.6) of all sponsorship (Table 3).

There was a small amount of variation in sponsorship over the three sporting levels, however the "gambling" category was consistently the most common, apart from "other". There was significantly more "alcohol" sponsorship at club level, compared to national levels (rate ratio (RR) = 3.77; 95% CI = 1.55, 9.20; p = 0.001). Also, significantly more "sporting organisations" had sponsorship at the national level compared to club levels (RR = 5.81; 95% CI = 2.65, 12.72, p < 0.001).

There was a high degree of variation between the sponsorship of different sports. There was significantly more "alcohol" sponsorship for rugby compared to all the other sports collectively (RR = 2.47; 95% CI = 1.60, 3.79; p < 0.001). Furthermore, there was significantly more "alcohol" sponsorship in male sports (ie, those only in the top five for males) compared to female sports (ie, those only in the top five for females) (RR = 1.83; 95% CI = 1.05, 3.18; p = 0.01). Also there were significantly more "unhealthy food" sponsors within the sport of touch rugby compared to other sports collectively (RR = 6.54; 95% CI = 2.07, 20.69; p = 0.01).

### Sponsor type

The two most common sponsor types were "franchise/multi-nationals" and "private companies" which made up 38.4% and 34.4% of total sponsorship listings on websites respectively (4). There were only small contributions from city council, central government and non-government agencies, which made up 3.3%, 2.8% and 2.7% of total sponsorship listings respectively. There were significantly more "private companies" sponsoring club level compared to national level teams (RR = 8.41; 95% CI = 4.06, 17.42, p < 0.001). Also there were significantly more "government agencies" and "franchise/multi-nationals" sponsoring national level teams compared to club ones (RR = 15.38; 95% CI = 4.52, 52.3, p < 0.001 and RR = 2.08; 95% CI = 1.69, 2.55, p < 0.001 respectively). Of the companies that specifically sponsored "junior" teams, clubs and tournaments there were significantly more "franchise/multi-nationals" companies, compared to all other sponsors (RR = 1.54; 95% CI = 1.13, 2.14; p = 0.01).

### Health-related classification

Of the sponsorship listings, 32.7% (95% CI = 29.1, 36.5) were classified as being linked to "unhealthy" products, whereas 15.5% (95% CI = 12.8, 18.6) were classified as being linked to "healthy" products (Table 5). There was significantly more "healthy" sponsorship at national compared to club levels (RR = 2.31; 95% CI = 1.54, 3.46, p < 0.001), and significantly more "healthy" sponsorship in basketball compared to other sports (RR = 3.17; 95% CI = 1.79, 5.62; p < 0.001). However, there was significantly more "unhealthy" sponsorship in touch rugby (RR = 1.64; 95% CI = 1.06, 2.55; p = 0.03).

### Sponsorship of junior sports

Out of the 640 sponsorship listings on websites, 33 were classified as sponsoring "junior" sports (ie, 5.2%). Within this grouping, there was significantly more "unhealthy food" sponsorship when compared to all other sponsorship (RR = 14.72, 95% CI = 6.22, 34.8; p < 0.001). On the Wellington junior touch rugby website, one fast food company even offered players a free refill of soft drink (itself classified as an "unhealthy food") if they brought their sports drink bottles into the restaurant.

### Naming rights of sponsors

A total of 24 sponsorship listings (3.8%) reported sponsor naming rights of either specific sports teams or specific tournaments. Out of these 24, 46% were in the "unhealthy" sponsorship category (7 gambling, 3 alcohol and 1 unhealthy food). Three were "healthy" and the remaining six were "not classified".

## Discussion

### Main findings and interpretation

This pilot study found that sponsorship of these popular sports was common (ie, a total of 640 sponsors over the three levels and the eight sports, in a total of 107 websites). The average number of sponsors was 8.2 per website. However, these figures could underestimate actual "real world" sponsorship levels, as some websites had no sponsorship information at all and not all clubs had functioning websites.

Of concern from a public health perspective was the imbalance between "healthy" and "unhealthy" sponsorship. Overall, the level of website sponsorship that was classified as "unhealthy", (32.7%) was over twice that which was classified as "healthy" (15.5%). The amount of "unhealthy" sponsorship is also probably an under-estimate, given that our classification system was conservative (eg, products promoting both inactive entertainment and non-active transport were not included in the "unhealthy" category). Furthermore, seven of the top ten most frequently listed sponsors were classified as being involved in "unhealthy" sponsorship (five gambling trusts, one brewery, and one fast food company).

A general principle of corporate sponsorship suggests that for a sponsorship deal to be successful commercially, there needs to be a good fit between the sponsor's target market and the event participants and followers [4]. In New Zealand, there has been a long-standing relationship between alcohol and rugby, and this could explain the higher prevalence of alcohol sponsorship for rugby [14]. This same principle may also explain the higher level of alcohol sponsorship in top male sports, as New Zealand males generally have a higher rate of alcohol consumption than females.

Furthermore, the advantages of "fit" could also explain the relatively higher prevalence of "unhealthy food" sponsorship within the sport of touch rugby given that touch is most popular in Maori and Pacific Island young people in New Zealand [12]. These two populations have high consumption patterns of "unhealthy foods" eg, the highest intakes of total and saturated fat, compared to New Zealand European children [15]. They also have relatively higher rates of obesity, compared to New Zealand European children [16].

Of sponsorship particularly targeting junior players and teams, there was a higher proportion of "unhealthy food" sponsorship. Young people offer a very attractive market for food companies since they influence their parents' spending, have considerable personal spending power of their own, and have a lifetime of spending ahead of them [17]. An added bonus of sponsoring junior teams is that it offers a way of getting young people and their parents into the sponsor's store and/or consuming their product, something that is important for some food retailers. An example of this was found on the Wellington junior touch website where a fast food company offered a free refill of soft drink to junior players, but only if they came into the company's restaurant.

Of all the sponsors, 24 had specific naming rights of teams or tournaments, with seven classified as gambling sponsors and three as alcohol sponsors. This is of particular concern, as naming rights offer these sponsors of "unhealthy" products not only commercial exposure but also the normalisation of their products. In particular, the relatively high proportion of naming rights agreements by gambling trusts appears to be a problem warranting further investigation by policy makers.

The largest categories of sponsorship were that supplied by multi-national/franchise companies (38.4%) and from private companies (19.1%). There were only small contributions from city councils (3.3%) and government agencies (2.8%). These former two company types, along with gambling trusts, make up all of the "unhealthy" sponsorship listings. Therefore, if there were to be any restrictions on "unhealthy" sponsorship there would probably need to be a much larger contribution from councils and government agencies to make up the gap in funding.

### Study limitations

As one component of this study was a pilot study of club and regional websites from only one region, the results for these organisational levels may not be generalisable to the rest of New Zealand. Furthermore, the study was limited to a cross-sectional design that cannot detect temporal patterns. For example, some of the sports examined were in the "off-season" at the time of sampling (summer in New Zealand) and so the sponsorship information may have not been recently updated for some sports.

As detailed above, the classification of "unhealthy" that was used was defined conservatively. In particular, the inactive entertainment and non-active transport categories were left out of the "unhealthy" category, even though they may contribute to lower energy expenditures and therefore obesity. Furthermore, the frequency of sponsorship listings was only based on the number of websites/teams, and included no consideration of the amount of money or other resources provided (eg, player-of-the-day vouchers, exclusive stocking agreements).

The validation study found an inter-rater reliability of 95% for the number of sponsors and 100% for further categorisations. This suggests that this aspect of the methodology was reasonably robust. Nevertheless, the small differences may have arisen due to the six week time delay between the full study and the validation study (ie, during which changes in sponsorship and/or website upgrades occurred – including the fixing of some websites that were initially unavailable due to technical problems).

### Research and policy implications

Given the limitations detailed above and the pilot nature of aspects of this study, it is clear that further methodological improvements could be pursued in future studies. In particular, New Zealand and other countries should consider sampling multiple regions to provide more robust overall baseline values for monitoring trends in sports sponsorship.

Other future research in this area may include examining other advertising and sponsorship associated with sports (eg, even including sponsorship messages on team members clothing). Furthermore, it may be useful to look at sponsorship from the perspective of the sponsoring company (eg, the expenditure in their annual reports and their statements on their own websites).

Despite the pilot nature of aspects of this study, it has provided some initial information about the imbalance between "unhealthy" and "healthy" sponsorship of popular New Zealand sports. These findings provide tentative support for responses by policy makers to reduce this imbalance. One possible response would be to restrict "unhealthy" sponsorship of sports (eg, as done successfully with the complete restrictions on tobacco sponsorship in New Zealand). Yet we acknowledge that this approach would require a greater level of political and societal acceptance of what sponsorship around food, alcohol and gambling can be defined as "unhealthy".

At the same time, sponsorship appears essential for keeping sports sustainable, and sponsorship money ideally should come from health promoting forms of sponsorship. For example, this was done by the government-funded Health Sponsorship Council in the 1990s in New Zealand, when tobacco sponsorship was eliminated. Another approach might be to create a government-managed "blind fund" so that "sponsorship" money is still supplied to sports clubs or teams but the supplier is not linked to a particular funder. This approach may be particularly relevant for gaming machine trusts that have a legal obligation in New Zealand to donate a proportion of their revenues to the community. Greater government control over the sponsorship could also allow sponsorship to be targeted towards those sports where the need is greatest (eg, sports favoured by those populations who are at greatest risk from obesity and diabetes). Priority could also be given to those sports that are likely to have the greatest long-term health benefits (eg, sports which are associated with lifelong physical activity such as athletics, swimming, cycling, tennis, golf etc) [18].

## Conclusion

This study found that the sponsorship of popular sports for young people is dominated by sponsorship associated with "unhealthy" products (ie, predominantly gambling, alcohol and unhealthy food) relative to sponsorship associated with "healthy" products. Furthermore, there seem to be some sports which have been targeted by "unhealthy" sponsorship, in particular alcohol sponsorship and rugby, and "unhealthy food" sponsorship and touch rugby. Health agencies in New Zealand and elsewhere should consider funding more detailed research on sports sponsorship that covers all regions and levels of sport. However, if similar results to this study are found then governments may need to consider regulations that limit "unhealthy" sponsorship and/or adopt alternative funding mechanisms for sponsoring popular sports.

## Competing interests

The author(s) declare that they have no competing interests.

## Authors' contributions

AM assisted in designing the study, managed the study, undertook the data collection, the data analysis and did the first drafts of the manuscript.

NW assisted in study design and management, data analysis and revising manuscript drafts.

LS assisted in study design and management and revising manuscript drafts.

GT assisted in study design and revising manuscript drafts.

All authors read and approved the final manuscript.

## Pre-publication history

The pre-publication history for this paper can be accessed here:



## Figures and Tables

**Table 1:** Websites for the top male and female sports (for young people) and levels of sponsorship

	Total no. of websites	No. of websites with sponsorship listings	No. of sponsor-ship listings	Average no. sponsorship listings per website*
***All websites***	107	79	640	8.1
				
***Organisational level***				
Club	86	58	406	7.0
Regional	12	12	106	8.8
National	9	9	131	14.6
				
***Sport***				
Athletics	29	11	38	3.4
Basketball	2	2	15	7.5
Cricket	12	11	74	6.7
Netball	10	9	83	9.2
Rugby	15	15	192	12.8
Soccer	28	22	166	7.6
Tennis	9	7	53	7.6
Touch	2	2	19	9.5

* For websites with any non-zero level of sponsorship (ie, excluding those with no accessible information about current sponsorship).

**Table 2:** Top 10 sponsors (by number of sponsorship listings on websites) for the top male and female sports (n = 398 different sponsors)

	Sponsorship listings		Main product/activity of the sponsor	Classification used in this study
	N	%		

NZ Community Trust	40	6.3	Gaming machine trust	Gambling
Wellington City Council	19	3	City council	Non-sporting organisation
Lion Foundation	17	2.7	Gaming machine trust	Gambling
Pub Charity	10	1.6	Gaming machine trust	Gambling
SPARC	10	1.6	Sporting body	Sporting organisation
Champs Sports	8	1.3	Sports brand	Sports goods
Scottwood Trust	8	1.3	Gaming machine trust	Gambling
Unison Trust	8	1.3	Gaming machine trust	Gambling
DB	7	1.1	Beer	Alcohol
McDonalds	6	0.9	Fast food chain	Unhealthy food

**Table 3:** Categorisation of sponsorship listings on websites for the top male and female sports for young people

	Unhealthy food		Alcohol		Gambling		Non-active Transport* & inactive entertainment**		Health promotion & healthy food		Sports goods		Sports organisations		Sports venues		All other†		Total
	N	%	N	%	N	%	N	%	N	%	N	%	N	%	N	%	N	%	N
***Level***																			
Club	9	2.2	58	14.4	73	18.1	14	3.5	2	0.4	28	6.9	9	2.2	5	1.2	206	51.1	403
Regional	4	3.8	9	8.5	19	17.9	8	7.6	1	0.9	11	10.4	1	0.9	9	8.5	44	41.5	106
National	5	3.8	5	3.8	28	21.4	14	10.7	3	2.3	13	9.9	17	13.0	0	0	46	35.1	131

***Sport***																			
Athletics	0	0	4	10.5	7	18.4	2	5.3	0	0	4	10.5	6	15.8	0	0	15	39.5	38
Basketball	1	6.7	0	0	1	6.7	2	13.4	0	0	3	20.0	4	26.7	0	0	4	26.7	15
Cricket	2	2.7	7	9.5	16	21.6	4	5.5	1	1.4	7	9.5	2	2.7	1	1.4	35	47.3	74
Netball	4	4.8	6	7.2	18	21.7	8	9.6	0	0	7	8.4	2	2.4	5	6.0	33	39.8	83
Rugby	3	1.6	37	19.3	25	13.0	9	4.7	1	0.5	8	4.2	4	2.1	5	2.6	100	52.1	192
Soccer	4	2.4	12	7.2	38	22.9	10	6.0	3	1.8	16	9.6	4	2.4	1	0.6	78	47.0	166
Tennis	1	1.9	5	9.4	9	17.0	1	1.9	0	0	4	7.5	4	7.5	1	1.9	28	52.8	53
Touch	3	15.8	1	5.3	6	31.6	0	0	1	5.3	3	15.8	1	5.3	1	5.3	3	15.8	19

***Junior***‡	8	24.2	1	3.0	5	15.2	0	0	1	3.0	1	3.0	1	3.0	0	0	16	48.5	33

All websites	18	2.8	72	11.3	120	18.8	38	5.6	6	0.9	52	8.1	27	4.2	14	2.2	296	46.3	640

* Sponsorship of products involving lower human energy output (eg, car use), compared to more physically active transport (eg, cycling).

** Sponsorship of products related to lower energy output (eg, cinema and video watching), compared to physically active entertainment (eg, mini-golf).

† All other includes: non-sporting organisations, "other food" (mixed, non-classified foods), and "other" groupings.

‡ Junior level sponsorship included those sponsorship listings that specifically mentioned sponsorship of junior clubs, junior teams or school-aged tournaments.

**Table 4:** Sponsorship listings on sports websites by sponsoring company or organisation

	City council		Franchise or multi-national		Gambling company/Trust		Government agency		Non-government agency*		Private company		Total
	N	%	N	%	N	%	N	%	N	%	N	%	N
***Level***													
Club	18	4.5	117	29	74	18.4	3	0.7	10	2.5	181	44.9	403
Regional	2	1.9	50	47.2	19	17.9	0	0	3	2.8	32	30.2	106
National	1	0.8	79	60.3	25	19.1	15	11.5	4	3.1	7	5.3	131
													
***Sport***													
Athletics	8	21.1	9	23.7	8	21.1	4	10.5	2	5.3	7	18.4	38
Basketball	0	0	10	66.7	1	6.7	3	20	1	6.7	0	0	15
Cricket	4	5.4	31	41.9	15	20.3	1	1.4	5	6.8	18	24.3	74
Netball	1	1.2	39	47	18	21.7	1	1.2	1	1.2	23	27.7	83
Rugby	2	1	75	39.1	25	13	0	0	4	2.1	86	44.8	192
Soccer	2	1.2	58	34.9	37	22.3	4	2.4	2	1.2	63	38	166
Tennis	4	7.5	16	30.2	8	15.1	3	5.7	2	3.8	20	37.7	53
Touch	0	0	8	42.1	6	31.6	2	10.5	0	0	3	15.8	19
													
***Junior***	2	6.1	19	57.6	5	15.2	1	3	0	0	6	18.2	33
													
***All websites***	21	3.3	246	38.4	118	18.4	18	2.8	17	2.7	220	34.4	640

* For example, sports-related organisations.

**Table 5:** Health aspects of sponsorship listings on websites for the top male and female sports for young people

	"Healthy"		"Unhealthy"		"Non Classified"*		All websites
	
	N	%	N	%	N	%	N
***Level***							
Club	44	10.9	139	34.5	220	54.6	403
Regional	22	20.8	32	30.2	52	49.1	106
National	33	25.2	38	29.0	60	45.8	131
							
***Sport***							
Athletics	10	26.3	11	28.9	17	44.7	38
Basketball	7	46.7	2	13.3	6	40.0	15
Cricket	11	14.9	24	32.4	39	52.7	74
Netball	14	16.9	28	33.4	41	49.4	83
Rugby	18	9.4	65	33.9	109	56.8	192
Soccer	24	14.5	54	32.5	88	53.0	166
Tennis	9	17.0	15	28.3	29	54.7	53
Touch	6	31.6	10	52.6	3	15.8	19
							
Junior**	3	9.1	14	42.4	16	48.5	33
							
***All websites***	99	15.5	209	32.7	332	51.9	640

* "Not Classified" includes all groupings not in "healthy" and "unhealthy". This included non-sporting organisations, inactive entertainment, non-active transport, foods with mixed nutrition aspects, and other categories.

** Junior level sponsorship included those sponsorship listings that specifically mentioned sponsorship of junior clubs, junior teams or school-aged tournaments.
